# Second-line paclitaxel in non-small cell lung cancer initially treated with cisplatin: a study by the European Lung Cancer Working Party

**DOI:** 10.1038/sj.bjc.6603772

**Published:** 2007-05-01

**Authors:** T Berghmans, J J Lafitte, J Lecomte, C G Alexopoulos, O Van Cutsem, V Giner, A Efremidis, M C Berchier, T Collon, A P Meert, A Scherpereel, V Ninane, N Leclercq, M Paesmans, J P Sculier

**Affiliations:** 1 Department of Critical Care & Thoracic Oncology, Institut Jules Bordet, 1, rue Héger-Bordet, B – 1000, Bruxelles, Belgium

**Keywords:** chemotherapy, sequential, advanced non-small cell lung cancer

## Abstract

In the context of a phase III trial comparing in advanced non-small cell lung cancer (NSCLC) sequential to conventional administration of cisplatin-based chemotherapy and paclitaxel, we evaluated the activity of paclitaxel as second-line chemotherapy and investigated any relation of its efficacy with the type of failure after cisplatin. Patients received three courses of induction GIP (gemcitabine, ifosfamide, cisplatin). Non-progressing patients were randomised between three further courses of GIP or three courses of paclitaxel. Second-line paclitaxel was given to patients with primary failure (PF) to GIP and to those progressing after randomisation to further GIP (secondary failure or SF). One hundred sixty patients received second-line paclitaxel. Response rates were 7.7% for PF and 11.6% for SF (*P*=0.42). Median survival times (calculated from paclitaxel start) were 4.1 and 7.1 months for PF and SF (*P*=0.002). In multivariate analysis, three variables were independently associated with better survival: SF (hazard ratio (HR)=1.55, 95% confidence interval (CI) 1.08–2.22; *P*=0.02), normal haemoglobin level (HR=1.56, 95% CI 1.08–2.26; *P*=0.02) and minimal weight loss (HR=1.79, 95% CI 1.26–2.55; *P*=0.001). Paclitaxel in NSCLC patients, whether given for primary or for SF after cisplatin-based chemotherapy, demonstrates activity similar to other drugs considered active as second-line therapy.

First-line cisplatin-based chemotherapy has demonstrated a palliative effect in advanced and metastatic non-small cell lung cancer (NSCLC) with a significant survival improvement, better symptoms control and reduced cost compared with supportive care only (Non Small Cell Lung Cancer Collaborative Group, 1995). Second-line chemotherapy with taxanes has also been shown effective (Barlesi *et al*, 2006; Sculier, 2006). Docetaxel, which has been shown to significantly improve survival compared with supportive care only (Shepherd *et al*, 2000), is currently proposed as second-line chemotherapy in NSCLC patients failing after platinum-based chemotherapy (Socinski *et al*, 2003; Pfister *et al*, 2004; European Lung Cancer Working Party, 2006). Paclitaxel, the other available taxane, has also demonstrated potential activity in the same indication in a few phase II studies (Socinski *et al*, 1999; Juan *et al*, 2002; Socinski *et al*, 2002; Sculier *et al*, 2002a; Buccheri and Ferrigno, 2004; Ceresoli *et al*, 2004; Yasuda *et al*, 2004). There is only one randomised phase II study, including 71 patients, which directly compares paclitaxel to docetaxel (Esteban *et al*, 2003).

In the present study, we studied all the patients included in a randomised phase III trial of the European Lung Cancer Working Party (ELCWP) who received paclitaxel as second-line chemotherapy. The primary aim of the above-mentioned phase III trial was to compare sequential administration of cisplatin-based chemotherapy followed by paclitaxel to a conventional cisplatin-based chemotherapy with paclitaxel as salvage therapy. The aim of the present study was the analysis of the activity and toxicity of paclitaxel when given as second-line chemotherapy in NSCLC patients, in PF or secondary failure (SF) after first-line cisplatin-based chemotherapy. Institutions that participated in the trials are set as Appendix section.

## PATIENTS AND METHODS

### Selection criteria

Eligibility criteria for registration in the study included:

- histologically or cytologically proven NSCLC
- previously untreated stage IV or stage IIIB with malignant pleural effusion patients
- Karnofsky performance status (PS) ⩾60

Other eligibility criteria were those previously published by the ELCWP (Sculier *et al*, 2002b).

### Treatment

Eligible patients received three courses of induction GIP (gemcitabine (1 g m^−2^ on days 1 and 8)+ifosfamide (3 g m^−2^ on day 1)+cisplatin (50 mg m^−2^ on day 1)) every 3 weeks. At evaluation, non-progressing patients (stable disease or objective responses) were randomised between three further similar courses of GIP or three courses of paclitaxel (225 mg m^−2^ over 3 h after appropriate antihypersensitivity premedication), every 3 weeks. Treatment was given until best response. Patients progressing after randomisation were treated with paclitaxel if in the GIP arm or GIP if in the paclitaxel arm. Paclitaxel at the above mentioned dosage was also given in case of disease progression (PD) after the three cycles of induction GIP.

Patients with failure to the induction GIP (primary failure or PF) and patients who progressed after randomisation to another three courses of GIP (SF) constitute the population of the present study.

The administration of a course of paclitaxel required that haematological (neutrophils >1500 mm^−3^ and platelets >100 000 mm^−3^) function had recovered. If the delay between two courses was more than 6 weeks, the patient went off-treatment. If neutrophil nadir was <500 mm^−3^ and platelet nadir <25 000 mm^−3^, dosage of paclitaxel was reduced to 75% of the previously administered dose.

### Criteria of evaluation

Patients were evaluated for response after the completion of three chemotherapeutic courses. Responses were assessed according to the usual criteria (Sculier *et al*, 2002b). Complete remission was defined as the disappearance of all signs of disease, for at least 4 weeks. In measurable disease, partial response (PR) was defined as ⩾50% decrease of the total tumour load in two observations not less than 4 weeks apart, in the absence of new lesions or progression in any existing lesion. Tumour load was estimated as the tumour area calculated by the multiplication of the longest diameter by the greatest perpendicular diameter. In assessable disease, PR was defined as an estimated decrease in tumour size of 50% or more. Progression (PG) was defined as an increase of ⩾25% in one or more measurable or assessable lesions or the appearance of new lesion(s). All other circumstances were classified as no change (NC). Early death due to PD before evaluation, toxic death due to chemotherapy or early chemotherapy discontinuation because of toxicity were considered as treatment failures and incorporated in the evaluable patients.

In addition, we defined stabilisation as either PR or NC. Survival was calculated from the start of second-line paclitaxel. WHO criteria were used to assess toxicity.

### Statistical methodology

Registration and randomisation were centrally performed by calling the ELCWP central office at the Jules Bordet Institute in Brussels. Survival curves were estimated using Kaplan–Meier method. The log rank test was used to compare survival curves. *P*-values for testing differences between proportions were calculated with *χ*^2^ tests or with Fischer's exact tests. A multivariate analysis for adjustment of the treatment effect taking into account prognostic factors was performed by fitting the data with a Cox model for duration of survival and a logistic regression model for objective response. The result of a statistical test was considered as significant when level of the *P*<0.05. All reported *P*-values are two-sided. Paclitaxel dose intensity (DI) was calculated as the ratio of the cumulative dose to the actual duration of treatment (and expressed in mg m^−2^ and per week). Relative DI was the ratio of the achieved DI divided by the planned DI that is 75 mg m^−2^ per week.

## RESULTS

A total of 493 patients were registered in the phase III trial between January 2000 and February 2004. Eight (1.6%) were ineligible. The principal characteristics at registration of the 485 eligible patients were previously published (Sculier *et al*, 2007). At the evaluation after GIP courses, PR, NC and PD were documented in 174, 115 and 123 patients, respectively. Of the 123 progressing after initial GIP chemotherapy patients (PF), 91 (74%) received paclitaxel as salvage treatment. Twenty-three had no further anticancer therapy, six received radiotherapy and three other chemotherapeutic regimens. Characteristics of the 91 patients at the time of second-line paclitaxel are shown in Table 1. Among the 140 patients randomised to further GIP, 69 (49%) received second-line paclitaxel at relapse (SF). Among those 69, 39 had responded and 30 demonstrated NC to the initial GIP chemotherapy. Their characteristics are also shown in Table 1.

Type of response in PF and SF groups is shown in Table 2. Objective response (OR) was documented in 15 patients: seven (7.7 %) in the PF group and eight (11.6%) in the SF group. NC was observed in 27 patients, nine (9.9%) in PF and 18 (26.1%) in SF. There was no significant (*P*=0.42) difference in OR between the two groups. On the contrary, in terms of stabilisation rate (OR+NC), the difference between the two groups reached significance (*P*=0.006). The results remained unchanged after adjustment for haemoglobinemia at the start of paclitaxel. In the SF group, we looked at the impact of the delay between the time of documentation of the first response or NC after the initial GIP (date of the third cycle of induction GIP plus 21 days) and the time of documentation of the first progression. The median time to progression (TTP) after the first GIP was 4 months (range 0.7–22.1 months). The SF group was divided according to the median TTP in an early progression group (SF1; TTP below the median) and a late progression group (SF2; TTP above the median). Three (9.1%) and five (13.9%) patients demonstrated PR in the SF1 and SF2 groups, respectively. Detailed results are shown in Table 2. There was no statistical difference in OR rate between PF and SF1 or SF2 groups (*P*=0.30). All the analyses were performed in an intent-to-treat level.

Considering patients' characteristics at the start of paclitaxel, no significant prognostic factor for response can be identified in univariate analysis (data not shown), with the exception of a favourable initial response to GIP. Patients with OR to GIP had higher OR rates to paclitaxel (18%) than those without (7%) (*P*=0.05). The same analysis was performed for the patients with disease stabilisation. The results are shown in Table 3. Significantly favourable prognostic factors were Karnofsky PS above 70 (*P*=0.03), normal haemoglobin level (*P*=0.001), objective response to initial GIP (*P*=0.003) and late SF (*P*=0.007). When we divided the SF group according to more standard delays such as 3 or 6 months, the same results were found. All the variables with a *P*<0.20 were included in a multivariate analysis. Two factors found to be predictive of stabilisation in patients receiving second-line paclitaxel: objective response to induction GIP (odds ratio 5.28, 95% confidence interval (CI) 2.01–13.87, *P*=0.001) and normal haemoglobin level (odds ratio 3.62, 95% CI 1.49–8.77, *P*=0.004).

**Figure 1:**
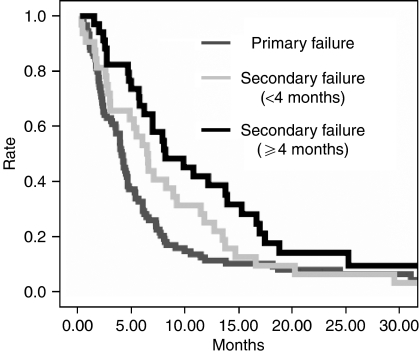
Survival curves of the patients with NSCLC treated with second-line paclitaxel.

Survival rates are shown in Table 4 and Figure 1. Four patients were lost to follow up and excluded from the survival analysis. At the time of the analysis, 144 patients were dead (85 out of 90 in PF, 31 out of 32 in SF1, 28 out of 34 in SF2). Median survival time (MST) was 4.1 months (95% CI 3.5–4.7 months) and 7.1 months (95% CI 5.3–8.9 months) for PF and SF group, respectively (hazard ratio (HR) 1.67, (95% CI 1.19–2.33; *P*=0.003). When SF patients were divided into early and late progression, MST were 6.5 (95% CI 4.9–8.0 months) and 8.2 months (95% CI 4.1–12.2 months), respectively. HR for the comparisons among the three groups were 1.35 for PF *vs* SF1 (95% CI 0.89–2.05, *P*=0.15), 2.01 for PF *vs* SF2 (95% CI 1.30–3.09, *P*=0.001) and 1.48 for SF1 *vs* SF2 (95% CI 0.89–2.46; *P*=0.14). Similar results were found when patients with SF were separately analysed according to a 3 or a 6 months interval between first OR documentation and first progression (data not shown).

Results of the prognostic factors analysis for survival are summarised in Table 5. In univariate analysis, variables significantly associated with better survival were: good PS (*P*=0.001), minimal weight loss (*P*<0.001), normal haemoglobin level (*P*=0.003), objective response to initial GIP (*P*=0.02) and progression free interval (PF *vs* SF, *P*=0.002). All these variables, excepting PS because of a very high rate of missing values, were selected for a Cox multivariate analysis. Three variables were independently associated with better survival: SF (HR=1.55, 95% CI 1.08–2.22; *P*=0.02), normal haemoglobin level (HR=1.56, 95% CI 1.08–2.26; *P*=0.02) and minimal weight loss (HR=1.79, 95% CI 1.26–2.55; *P*=0.001).

Data on dates and doses of paclitaxel courses were available for 148 out of 160 (92.5%) patients. Among them, 99 received at least three courses and 23 received at least six courses. There were few delayed administrations (5%) and dose reductions (4%). Calculated in the 99 patients who received at least three courses, the DI ranged between 47 and 83 mg m^−2^ week^−1^ (median 70) and the median relative DI was 94%. Similar results were obtained when the analysis was performed in all 148 patients.

Toxicity was minimal. Twelve and 20 patients were not assessable for non-haematological and haematological toxicities, respectively. Grade III–IV non-haematological toxicity comprised peripheral neuropathy (6%), infection (4%), cardiac (1%), nausea, skin reaction and diarrhoea (<1% each). Grade III–IV leucopenia and thrombopenia were observed in 11 and <1% of the patients, respectively.

## DISCUSSION

We found that paclitaxel is an active drug in both PF and SF, although response rates and survival are better in SF patients, especially in those with late SF.

Our study addressing, in fact, the question of the effectiveness of paclitaxel as second-line chemotherapy in patients with advanced NSCLC who failed first-line cisplatin-containing chemotherapy presents some interesting peculiarities:

1. Paclitaxel as second-line chemotherapy was part of the design of a prospective randomised phase III trial. Therefore, the majority of progressing/relapsing patients could tolerate high dose of paclitaxel (225 mg m^−2^) every 3 weeks as it was predicted in the protocol.
2. In variance with other second-line phase II studies, patients population in our study was homogeneous in terms of previously administered chemotherapy, as all had received the same initial cisplatin-based regimen.
3. Unlike in other studies, the patients who received paclitaxel were not selected at the time of second-line treatment, thus, allowing a better characterisation of the type of patients who could benefit from second-line paclitaxel.

Therefore, our findings can be considered more relevant information for implementation in clinical practice.

The main criticism of our study would be the decision to use paclitaxel instead of docetaxel as second-line chemotherapy. Nevertheless, at the time we designed our prospective phase III trial, there were no published randomised studies confirming the superiority of docetaxel *vs* best supportive care for relapsing NSCLC patients. As both paclitaxel and docetaxel had demonstrated interesting response rates as second-line treatment, in phase II studies, and because docetaxel was not yet broadly marketed, we decided to use paclitaxel, being the only drug available to all participating centres of the ELCWP.

It is of interest that the results observed with paclitaxel in phase II studies compared well with those obtained with docetaxel in randomised phase II and III trials. Response rates with paclitaxel of the range of 0–38% in previously published studies (Socinski *et al*, 1999, 2002; Juan *et al*, 2002; Sculier *et al*, 2002a; Buccheri and Ferrigno, 2004; Ceresoli *et al*, 2004; Yasuda *et al*, 2004), and of the order of 7.7 and 11.6% in PF and SF patients, respectively, observed in the present study are similar to those with docetaxel, ranging from 2.7 to 12.6% (Fossella *et al*, 2000; Shepherd *et al*, 2000; Gridelli *et al*, 2004; Hanna *et al*, 2004; Quoix *et al*, 2004; Gervais *et al*, 2005; Schuette *et al*, 2005; Camps *et al*, 2006).

Furthermore, survival with second-line paclitaxel in our study, significantly better for SF patients (7.1 months) than PF ones (4.1 months), was of the same magnitude than with second-line docetaxel in published randomised studies, reporting median survival rates (MST) ranging from 4.7 to 9.2 months (Fossella *et al*, 2000; Shepherd *et al*, 2000; Gridelli *et al*, 2004; Hanna *et al*, 2004; Quoix *et al*, 2004; Gervais *et al*, 2005; Schuette *et al*, 2005; Camps *et al*, 2006). Similar were MST with paclitaxel in previous studies, ranging from 4.5 to 14 months (Socinski *et al*, 1999, 2002; Juan *et al*, 2002; Sculier *et al*, 2002a; Buccheri and Ferrigno, 2004; Ceresoli *et al*, 2004; Yasuda *et al*, 2004). The equivalence of both drugs was evaluated in one small randomised phase II study only. No statistically significant difference was found in terms of response rate (14 *vs* 3%) and median survival (105 *vs* 184 days) (Esteban *et al*, 2003).

The most important finding of our study was the identification of three groups of patients with distinct prognosis. Patients failing during the initial cisplatin chemotherapy presented with the poorest response rate and survival. Patients with SF could be divided, according to the delay between the documentation of stabilisation (PR or NC) to initial cisplatin and relapse, into a short and a long delay group. The importance of treatment-free interval is well known in small cell lung cancer with higher probability to achieve a response with second-line chemotherapy if the treatment free interval is in excess of 3 months (Postmus, 2005). We also found that survival was influenced by progression-free interval in advanced NSCLC patients when comparing among PF, SF1 and SF2 groups.

Related toxicity is an important parameter in deciding among drugs with similar activity which one to use. We observed infrequent grade III/IV toxicity associated with paclitaxel. Non-haematological toxicities were of the same magnitude in the three biggest docetaxel trials (Fossella *et al*, 2000; Shepherd *et al*, 2000; Hanna *et al*, 2004) but more profound neutropenia were described with docetaxel.

We observed that stabilisation rates and survival were influenced by haemoglobin level. Haemoglobinemia can be influenced by various factors like transfusion policy, use of erythropoietin or time since end of platinum infusion. Haemoglobin levels used in the prognostic factor analysis were measured at the time or near before the first infusion of paclitaxel given as second-line therapy. We cannot definitively exclude that previous transfusions or prior erythropoietin administration influenced haemoglobin level at this time. However, in our analysis, we took into account time since end of platinum-based chemotherapy by considering the covariate type of failure (PF vs SF after a short or long delay) and haemoglobinemia remained a significant factor after adjustment for type of failure. This means that even if the level of haemoglobin is correlated to the type of failure, it brings some useful additional independent information to explain further survival.

In conclusion, paclitaxel is a well-tolerated and active drug for second-line therapy in patients with NSCLC failing or relapsing after cisplatin-based chemotherapy. Although all progressing patients could potentially benefit from paclitaxel use, we were able to define a group with better prognosis. Patients with stabilised disease (PR or NC) on initial cisplatin who relapsed after a long progression-free interval have a higher likelihood to respond to second-line paclitaxel and to have longer survival. Furthermore, response and survival rates with paclitaxel seems to be similar to those with docetaxel, although this needs to be confirmed in a prospective randomised phase III trial.

## Figures and Tables

**Table 1:** Characteristics at registration of the patients at time of second-line paclitaxel

	**PF**	**SF**	
***N***	**91**	**69**	***P*-value**
*Gender*			
Male	77 (85%)	52 (75%)	0.16
Female	14 (15%)	17 (25%)	
			
*Age*			
<60 years	50 (55%)	40 (58%)	0.75
⩾60 years	41 (45%)	29 (42%)	
			
*Histology*			
Squamous	14 (15%)	19 (27%)	0.10
Adenocarcinoma	55 (60%)	40 (58%)	
Other	22 (24%)	10 (15%)	
			
*Performance status*			
⩽70	35 (44%)	18 (38%)	0.58
80–100	44 (56%)	29 (62%)	
Unknown	12	22	
			
*Type of lesion*			
Assessable	25 (28%)	15 (22%)	0.46
Measurable	66 (72%)	54 (78%)	
			
*Stage*			
III	3 (3%)	1 (1%)	0.63
IV	87 (97%)	68 (99%)	
Unknown	1	0	
			
*Weight loss* a			
<5%	48 (53%)	39 (64%)	0.24
⩾5%	42 (47%)	22 (36%)	
Unknown	1	8	
			
*Haemoglobinemia*			
12–18 g dl^−1^	22 (25%)	30 (49%)	0.003
<12 g dl^−1^	66 (75%)	31 (51%)	
Unknown	3	8	
			
*Response to initial GIP*			
PR	—	39	—
NC	—	30	
PD	91	—	

NC=no change; PD=progressive disease; PF=primary failure; PR=partial response; SF=secondary failure.

a Weight loss between first induction GIP and first paclitaxel infusion.

**Table 2:** Response rates to second-line paclitaxel in patients with non-small cell lung cancer

	**PF**	**SF**	**SF1**	**SF2**
*N*	91 (57%)	69 (43%)	33	36
Partial response	7 (7.7%)	8 (11.6%)	3 (9.1%)	5 (13.9%)
No change	9 (9.9%)	18 (26.1%)	9 (27.3%)	9 (25%)
Progression	61 (67%)	31 (44.9%)	15 (45.5%)	16 (44.4%)
Early death malignant disease	8 (8.8%)	5 (7.2%)	4 (12.1%)	1 (2.8%)
Toxic death	1 (1.1%)	1 (1.4%)	1 (3%)	—
High toxicity	4 (4.4%)	1 (1.4%)	—	1 (2.8%)
Unassessable	1 (1.1%)	5 (7.2%)	1 (3%)	4 (11.1%)

PF=primary failure; SF=secondary failure; SF1=early secondary failure; SF2=late secondary failure.

**Table 3:** Univariate analysis of prognostic factors determining stabilisation with second-line paclitaxel

	**Clinical benefit**	***P*-value**
*Gender*		
Male	32/129 (25%)	0.50
Female	10/31 (32%)	
		
*Age*		
<60 years	20/90 (22%)	0.21
⩾60 years	22/70 (31%)	
		
*Histology*		
Squamous	9/33 (27%)	0.94
Adenocarcinoma	24/95 (25%)	
Other	9/32 (28%)	
		
*Karnofsky*		
⩽70	9/53 (17%)	0.03
80–100	26/73 (36%)	
		
*Type of lesion*		
Assessable	8/40 (20%)	0.41
Measurable	34/120 (28%)	
		
*Weight loss* a		
<5%	28/87 (32%)	0.14
⩾5%	13/64 (20%)	
		
*Haemoglobinemia*		
12–18 g dl^−1^	23/52 (44%)	0.001
<12 g dl^−1^	18/97 (19%)	
		
*Response to GIP*		
Objective response	18/39 (46%)	0.003
No objective response	24/121 (20%)	
		
*Type of failure*		
Primary failure	16/91 (18%)	0.007
SF1	12/33 (36%)	
SF2	14/36 (39%)	

GIP=gemcitabine, ifosfamide, cisplatin; SF1=early secondary failure; SF2=late secondary failure.

a Weight loss between first induction GIP and first paclitaxel infusion.

**Table 4:** Survival rates of patients with non-small cell lung cancer treated with second-line paclitaxel

	**PF**	**SF**		***P*-value**
MST	4.1 months (95% CI: 3.5–4.7)	7.1 months (95% CI: 5.3–8.9)		0.002
	HR 1.67 (95% CI: 1.19–2.33)			0.003
				
		SF1	SF2	
MST	4.1 months	6.5 months	8.2 months	0.004
		HR 1.48 (95% CI: 0.89–2.46)		0.14

CI=confidence interval; HR=hazard ratio; MST=median survival time; PF=primary failure; SF=secondary failure; SF1=early secondary failure; SF2=late secondary failure.

**Table 5:** Univariate analysis of prognostic factors for survival in patients receiving second-line paclitaxel

	***N* patients/ *N* events**	**MST (months)**	***P*-value**
*Gender*			
Male	126/116	4.8	0.30
Female	30/28	6.5	
			
*Age*			
<60 years	88/78	5.0	0.95
⩾60 years	68/66	5.3	
			
*Type of lesions*			
Assessable	38/34	5.5	0.92
Measurable	118/110	4.9	
			
*Histology*			
Squamous	31/29	6.2	0.70
Adenocarcinoma	93/84	4.6	
Other	32/31	4.7	
			
*Karnofsky*			
⩽70	52/50	3.9	0.001
80–100	73/66	6.6	
			
*Weight loss* a			
<5%	87/77	6.1	<0.001
⩾5%	63/62	3.9	
			
*Haemoglobinemia*			
12–18 g dl^−1^	52/46	8.0	0.003
<12 g dl^−1^	96/91	4.3	
			
*Response to GIP*			
Objective response	37/32	7.9	0.02
No objective response	119/112	4.5	
			
*Progression-free interval*			
Primary failure	85/90	4.1	0.002
Secondary failure	59/66	7.1	

GIP=gemcitabine, ifosfamide, cisplatin; MST=median survival time.

a Weight loss between first induction GIP and first paclitaxel infusion.

## Appendix

The following institutions participated in the trials:

Institut Jules Bordet, Brussels, Belgium (JP Sculier, T Berghmans, AP Meert, P Van Houtte, N Leclercq, M Paesmans, P Mommen)

CHRU Calmette, Lille, France (JJ Lafitte, A Scherpereel)

CHU de Charleroi, Charleroi, Belgium (J Lecomte, J Thiriaux)

Hellenic Cancer Institut, St Savas Hospital, Athens, Greece (A Efremidis, G Koumakis)

Clinique Saint-Luc, Bouge, Belgium (O Van Cutsem)

Hospital de Sagunto, Valencia, Spain (V Giner)

Evangelismos General Hospital, Athens, Greece (CG Alexopoulos, M Vaslamatzis)

Hôpital de Hayange, Hayange, France (MC Berchier, P Botrus)

CHI Le Raincy, Montfermeil, France (T Collon)

CHU Saint-Pierre, Brussels, Belgium (V Ninane, R Sergysels, T Bosschaerts)

CH de Douai, Douai, France (MC Florin, E Maetz, S Desurmont)

CHR St-Joseph-Warquiginies, Boussu, Belgium (M Richez)

CH de Roubaix, Hôpital Victor Provo, Roubaix, France (F Kroll, F Steenhouwer, F Salez, A Strecker)

Hôpital Ambroise Paré, Mons, Belgium (P Wackenier, S Holbrechts)

CHR Saint-Joseph, Mons, Belgium (P Recloux)

CHG de Tourcoing, Tourcoing, France (X Ficheroulle)

CHU André Vésale, Montigny-le-Tilleul, Belgium (D Brohée)

Hôpital Erasme, Brussels, Belgium (S Luce, F Branle)

RHMS, IMC Tournai, Belgium (A Tagnon)

RHMS, Clinique Louis Caty, Baudour, Belgium (V Richard, D Diana, B Vanderschueren)

CH Peltzer-La Tourelle, Verviers, Belgium (JL Corhay, I Louviaux)

Clinique Teissier, Valenciennes, France (G Demarcq, F Radenne)

Cabinet Médical Dampierre, Anzin, France (JP Roux, B Stach)

CH d’Arras, Arras, France (JP Bervar)

Hôpital Brugmann, Brussels, Belgium (A Drowart, T Prigogine)

CH Dr Schaffner ‘Ameuille’, Lens, France (J Amourette, C Bergoin)

CH Etterbeek-Ixelles, Brussels, Belgium (G Plat)

CHI Poissy-St-Germain, St-Germain-en-Laye, France (S Jouveshomme)

## References

[bib1] Barlesi F, Jacot W, Astoul P, Pujol JL (2006) Second-line treatment for advanced non-small cell lung cancer: a systematic review. Lung Cancer 51: 159–172 16360238 10.1016/j.lungcan.2005.08.017

[bib2] Buccheri G, Ferrigno D (2004) Second-line weekly paclitaxel in patients with inoperable non-small cell lung cancer who fail combination chemotherapy with cisplatin. Lung Cancer 45: 227–236 15246195 10.1016/j.lungcan.2004.01.011

[bib3] Camps C, Massuti B, Jimenez A, Maestu I, Gomez RG, Isla D, Gonzalez JL, Almenar D, Blasco A, Rosell R, Carrato A, Vinolas N, Batista N, Giron CG, Galan A, Lopez M, Blanco R, Provencio M, Diz P, Felip E (2006) Randomized phase III study of 3-weekly versus weekly docetaxel in pretreated advanced non-small-cell lung cancer: a Spanish Lung Cancer Group trial. Ann Oncol 17: 467–472 16371411 10.1093/annonc/mdj115

[bib4] Ceresoli GL, Gregorc V, Cordio S, Bencardino KB, Schipani S, Cozzarini C, Bordonaro R, Villa E (2004) Phase II study of weekly paclitaxel as second-line therapy in patients with advanced non-small cell lung cancer. Lung Cancer 44: 231–239 15084388 10.1016/j.lungcan.2003.11.006

[bib5] Esteban E, Gonzalez de SL, Fernandez Y, Corral N, Fra J, Muniz I, Vieitez JM, Palacio I, Fernandez JL, Estrada E, Lacave AJ (2003) Prospective randomised phase II study of docetaxel versus paclitaxel administered weekly in patients with non-small-cell lung cancer previously treated with platinum-based chemotherapy. Ann Oncol 14: 1640–1647 14581272 10.1093/annonc/mdg456

[bib6] European Lung Cancer Working Party (2006) Traitement des cancers bronchiques non à petites cellules, non métastatiques et non résécable. Recommandations de pratique clinique de l'ELCWP. Rev Med Brux 27: 152–161 16894953

[bib7] Fossella FV, DeVore R, Kerr RN, Crawford J, Natale RR, Dunphy F, Kalman L, Miller V, Lee JS, Moore M, Gandara D, Karp D, Vokes E, Kris M, Kim Y, Gamza F, Hammershaimb L (2000) Randomized phase III trial of docetaxel versus vinorelbine or ifosfamide in patients with advanced non-small-cell lung cancer previously treated with platinum-containing chemotherapy regimens. The TAX 320 Non-Small Cell Lung Cancer Study Group. J Clin Oncol 18: 2354–2362 10856094 10.1200/JCO.2000.18.12.2354

[bib8] Gervais R, Ducolone A, Breton JL, Braun D, Lebeau B, Vaylet F, Debieuvre D, Pujol JL, Tredaniel J, Clouet P, Quoix E (2005) Phase II randomised trial comparing docetaxel given every 3 weeks with weekly schedule as second-line therapy in patients with advanced non-small-cell lung cancer (NSCLC). Ann Oncol 16: 90–96 15598944 10.1093/annonc/mdi018

[bib9] Gridelli C, Gallo C, Di MM, Barletta E, Illiano A, Maione P, Salvagni S, Piantedosi FV, Palazzolo G, Caffo O, Ceribelli A, Falcone A, Mazzanti P, Brancaccio L, Capuano MA, Isa L, Barbera S, Perrone F (2004) A randomised clinical trial of two docetaxel regimens (weekly vs 3 week) in the second-line treatment of non-small-cell lung cancer. The DISTAL 01 study. Br J Cancer 91: 1996–2004 15558071 10.1038/sj.bjc.6602241 PMC2409790

[bib10] Hanna N, Shepherd FA, Fossella FV, Pereira JR, De MF, von PJ, Gatzemeier U, Tsao TC, Pless M, Muller T, Lim HL, Desch C, Szondy K, Gervais R, Shaharyar, Manegold C, Paul S, Paoletti P, Einhorn L, Bunn Jr PA (2004) Randomized phase III trial of pemetrexed versus docetaxel in patients with non-small-cell lung cancer previously treated with chemotherapy. J Clin Oncol 22: 1589–1597 15117980 10.1200/JCO.2004.08.163

[bib11] Juan O, Albert A, Ordono F, Casany R, Caranana V, Campos JM, Alberola V (2002) Low-dose weekly paclitaxel as second-line treatment for advanced non-small cell lung cancer: a phase II study. Jpn J Clin Oncol 32: 449–454 12499416 10.1093/jjco/hyf098

[bib12] Non Small Cell Lung Cancer Collaborative Group (1995) Chemotherapy in non-small cell lung cancer: a meta-analysis using updated data on individual patients from 52 randomised clinical trials. Non-small Cell Lung Cancer Collaborative Group. BMJ 311: 899–909 7580546 PMC2550915

[bib13] Pfister DG, Johnson DH, Azzoli CG, Sause W, Smith TJ, Baker Jr S, Olak J, Stover D, Strawn JR, Turrisi AT, Somerfield MR (2004) American Society of Clinical Oncology treatment of unresectable non-small-cell lung cancer guideline: update 2003. J Clin Oncol 22: 330–353 14691125 10.1200/JCO.2004.09.053

[bib14] Postmus PE (2005) Second-line for small cell lung cancer: how-to-do-it? Lung Cancer 48: 263–265 15829327 10.1016/j.lungcan.2004.12.009

[bib15] Quoix E, Lebeau B, Depierre A, Ducolone A, Moro-Sibilot D, Milleron B, Breton JL, Lemarie E, Pujol JL, Brechot JM, Zalcman G, Debieuvre D, Vaylet F, Vergnenegre A, Clouet P (2004) Randomised, multicentre phase II study assessing two doses of docetaxel (75 or 100 mg m2^−1^) as second-line monotherapy for non-small-cell lung cancer. Ann Oncol 15: 38–44 14679117 10.1093/annonc/mdh005

[bib16] Schuette W, Nagel S, Blankenburg T, Lautenschlaeger C, Hans K, Schmidt EW, Dittrich I, Schweisfurth H, von Weikersthal LF, Raghavachar A, Reissig A, Serke M (2005) Phase III study of second-line chemotherapy for advanced non-small-cell lung cancer with weekly compared with 3-weekly docetaxel. J Clin Oncol 23: 8389–8395 16293869 10.1200/JCO.2005.02.3739

[bib17] Sculier JP (2006) CBNPC stade IV ‘Quel est le meilleur traitement de la rechute?’ Cours du Groupe d'Oncologie Thoracique de Langue Française (GOLF) Limoges 2006. Rev Mal Respir 23: 16S78–16S83 17268341

[bib18] Sculier JP, Berghmans T, Lafitte JJ, Richez M, Recloux P, Van CO, Ninane V, Mommen P, Paesmans M, Klastersky J (2002a) A phase II study testing paclitaxel as second-line single agent treatment for patients with advanced non-small cell lung cancer failing after a first-line chemotherapy. Lung Cancer 37: 73–77 12057870 10.1016/s0169-5002(02)00037-5

[bib19] Sculier JP, Lafitte JJ, Lecomte J, Alexopoulos CG, Van CO, Giner V, Efremidis A, Berchier MC, Collon T, Meert AP, Scherpereel A, Ninane V, Paesmans M, Berghmans T (2007) A phase III randomised trial comparing sequential chemotherapy using cisplatin-based regimen and paclitaxel to cisplatin-based chemotherapy alone in advanced non-small cell lung cancer. Ann Oncol doi: 10.1093/annonc/mdm084; [E-pub ahead of print] 10.1093/annonc/mdm084 17404152

[bib20] Sculier JP, Lafitte JJ, Lecomte J, Berghmans T, Thiriaux J, Florin MC, Efremidis A, Alexopoulos CG, Recloux P, Ninane V, Mommen P, Paesmans M, Klastersky J (2002b) A three-arm phase III randomised trial comparing combinations of platinum derivatives, ifosfamide and/or gemcitabine in stage IV non-small-cell lung cancer. Ann Oncol 13: 874–882 12123332 10.1093/annonc/mdf154

[bib21] Shepherd FA, Dancey J, Ramlau R, Mattson K, Gralla R, O'Rourke M, Levitan N, Gressot L, Vincent M, Burkes R, Coughlin S, Kim Y, Berille J (2000) Prospective randomized trial of docetaxel versus best supportive care in patients with non-small-cell lung cancer previously treated with platinum-based chemotherapy. J Clin Oncol 18: 2095–2103 10811675 10.1200/JCO.2000.18.10.2095

[bib22] Socinski MA, Morris DE, Masters GA, Lilenbaum R (2003) Chemotherapeutic management of stage IV non-small cell lung cancer. Chest 123: 226S–243S 12527582 10.1378/chest.123.1_suppl.226s

[bib23] Socinski MA, Schell MJ, Bakri K, Peterman A, Lee JH, Unger P, Yates S, Hudgens S, Kies MS (2002) Second-line, low-dose, weekly paclitaxel in patients with stage IIIB/IV nonsmall cell lung carcinoma who fail first-line chemotherapy with carboplatin plus paclitaxel. Cancer 95: 1265–1273 12216094 10.1002/cncr.10835

[bib24] Socinski MA, Steagall A, Gillenwater H (1999) Second-line chemotherapy with 96-hour infusional paclitaxel in refractory non-small cell lung cancer: report of a phase II trial. Cancer Invest 17: 181–188 10099656 10.3109/07357909909021419

[bib25] Yasuda K, Igishi T, Kawasaki Y, Kato K, Matsumoto S, Katayama S, Sako T, Shigeoka Y, Suyama H, Sugitani A, Yamamoto M, Hitsuda Y, Shimizu E (2004) Phase II study of weekly paclitaxel in patients with non-small cell lung cancer who have failed previous treatments. Oncology 66: 347–352 15331920 10.1159/000079481

